# Chitin Nanocomposite Based on Plasticized Poly(lactic acid)/Poly(3-hydroxybutyrate) (PLA/PHB) Blends as Fully Biodegradable Packaging Materials

**DOI:** 10.3390/polym14153177

**Published:** 2022-08-03

**Authors:** Magdalena L. Iglesias-Montes, Michelina Soccio, Valentina Siracusa, Massimo Gazzano, Nadia Lotti, Viviana P. Cyras, Liliana B. Manfredi

**Affiliations:** 1 Instituto de Investigaciones en Ciencia y Tecnología de Materiales, Facultad de Ingeniería, Universidad Nacional de Mar del Plata—Consejo de Investigaciones Científicas y Técnicas, Mar del Plata 7600, Argentina; mliglesias@fi.mdp.edu.ar (M.L.I.-M.); vpcyras@fi.mdp.edu.ar (V.P.C.); 2 Department of Civil, Chemical, Environmental and Materials Engineering, University of Bologna, 40131 Bologna, Italy; nadia.lotti@unibo.it; 3 Interdepartmental Center for Industrial Research on Advanced Applications in Mechanical Engineering and Materials Technology, CIRI-MAM, University of Bologna, 40126 Bologna, Italy; 4 Chemical Science Department, University of Catania, Viale A. Doria 6, 95125 Catania, Italy; vsiracus@dmfci.unict.it; 5 Institute of Organic Synthesis and Photoreactivity, National Research Council, 40129 Bologna, Italy; massimo.gazzano@isof.cnr.it; 6 Interdepartmental Center for Agro-Food Research, CIRI-AGRO, University of Bologna, 40126 Bologna, Italy

**Keywords:** poly(lactic acid), poly(hydroxybutyrate), chitin nanoparticles, nanocomposites, biodegradable polymers, packaging

## Abstract

Fully bio-based poly(lactic acid) (PLA) and poly(3-hydroxybutyrate) (PHB) blends plasticized with tributyrin (TB), and their nanocomposite based on chitin nanoparticles (ChNPs) was developed using melt mixing followed by a compression molding process. The combination of PHB and ChNPs had an impact on the crystallinity of the plasticized PLA matrix, thus improving its oxygen and carbon dioxide barrier properties as well as displaying a UV light-blocking effect. The addition of 2 wt% of ChNP induced an improvement on the initial thermal degradation temperature and the overall migration behavior of blends, which had been compromised by the presence of TB. All processed materials were fully disintegrated under composting conditions, suggesting their potential application as fully biodegradable packaging materials.

## 1. Introduction

Several biodegradable bio-based polymers have attracted special interest in the packaging industry since they have comparable properties to those of conventional polymers and allow for a reduction environmental impact [1]. A number of biopolymers are currently available on the market, including poly(lactic acid) (PLA) and poly(3-hydroxybutyrate) (PHB)—two of the most attractive commercial biopolymers due to a number of promising properties suitable for food packaging applications. Furthermore, over the last twenty years, plasticized blends of PLA-PHB have been extensively studied by reason of their improvement in the final blend properties when both polymers are mixed, that is, the occurrence of a synergistic effect [2,3,4,5,6].

The addition of nanoparticles into the polymeric packaging material makes it possible to modulate the physicochemical properties and leads to improvements in specific features such as barrier capability, mechanical resistance, and thermal stability [7]. In particular, in the development of packaging materials, the use of nanoparticles to improve barrier performance is interesting since they can create more tortuous paths for small molecules, such as gases and vapors [8].

Nano-fillers can be classified according to their origin (natural, semi-synthetic, or synthetic) [9]. In this sense, for formulations of biopolymeric nanocomposites, the use of nano-fillers of a natural origin is preferred in order to preserve the characteristics of bio-based and biodegradable materials [10].

The use of polysaccharide nanoparticles has attracted considerable attention in a wide range of areas and especially in packaging applications. Polysaccharides are the most abundant macromolecules in the biosphere and have remarkable features such as biocompatibility, biodegradability, renewable origin, and facile modification [11]. These complex carbohydrates, formed by monosaccharides linked by glycosidic bonds, are one of the main structural elements of plants and of animals’ exoskeletons (for example, cellulose, lignin, chitosan, chitin, etc.) or play a key role in the energy storage of plants (e.g., starch, paramylon, etc.) [12].

In the particular case of the production of films based on PLA-PHB, where the use of plasticizers is required to modulate the compatibility and flexibility of the blend, the barrier performance is usually affected by the presence of the plasticizer. Therefore, strategies such as the development of nanocomposites can be used to obtain materials that have a good balance between structural and functional properties.

Scientific articles of PLA-PHB polymeric matrices reinforced with cellulose nanocrystals (CNC) have been published [13,14,15,16], revealing a slight increase in the elastic modulus and tensile strength but without significant changes in the gas barrier properties or thermal stability. This indicates that much remains to be investigated in this field.

Chitin is the second most abundant polysaccharide in nature after cellulose, with global reserves of 100 billion tons [17]. It is widely distributed in the animal and plant kingdoms, constituting an important renewable resource. It is part of the skeletal structure of many invertebrates such as arthropods, mollusks, and annelids. It is also found in the structural tissue of some species of fungi and algae. Commercial chitin is extracted from crustacean waste from the fishing industry, the main sources being the exoskeletons of shrimp, crabs, lobsters, prawns, and krill [18]. Globally, approximately 6 million tons of crustaceans are discarded per year [19]. Despite its great abundance, chitin was, for a long time, an underutilized resource, compared to other polysaccharides (including chitosan, which is a derivative from chitin), due to its insoluble character.

It is a fairly recent trend that has embraced the importance of chitin as a promising source of new materials, especially as a nanostructured material in the form of chitin nanocrystals (ChNC) and/or nanofibers (ChNF) [20]. One of the first scientific papers in which this was described examined the use of ChNC as a new ecological nano-reinforcement in thermoplastic materials, and was published by Paillet and Dusfrene in 2001 [21].

Different techniques have been reported to prepare chitin nanoparticles (ChNP) with different morphologies [20], although the most widely used is acid hydrolysis, generally with hydrochloric acid (HCl). The important advantages of nanochitin such as non-toxicity, low density, insolubility in water, biodegradability, biocompatibility, easy surface modification, and especially its antimicrobial activity, favor its use in wide areas such as nanocomposites, food packaging, cosmetics, drug administration, and tissue engineering [22]. However, the exploitation of chitin nanoparticles as a route to manufacture nanocomposites with high performance and specific functionalities, in particular because of their attractive antifungal and barrier properties, remains a vast and largely unexplored field of research. ChNP have been used as nano-reinforcement of various polymers such as starch [23,24], carboxymethylcellulose [22], chitosan [25], PVOH [26], PLA [19,27,28], and PBAT [29]. Particularly, promising biodegradable material for food packaging based on PVA has been developed. Oyeoka et al. [30] found that the water vapor permeability and moisture uptake of PVA-gelatin matrix films decreased with the addition of CNC. On the other hand, Zhang et al. [31] reported on the optimized formulation of the silica-reinforced film of polyvinyl alcohol/liquefied ball-milled chitin (PVA/LBMC), which showed the best mechanical property, thermal stability, and preservation performance. Still, there is very little published information on the use of nanochitin in biodegradable polymer blends for systems similar to those studied in this work [32].

The purpose of the present study was to evaluate the role of chitin nanoparticles on the structural and functional properties of thermoplastic nanocomposite PLA-PHB-based films prepared by melt mixing and a compression molding approach. A number of previous works describing the PLA-PHB system have already been published [33,34,35]. The overall migration of films in the different simulants was also determined in order to know how the film fits the legal limits as a function of food polarity. Finally, the disintegrability of the films in composting conditions was tested to determine their post-use opportunities.

## 2. Materials and Methods

### 2.1. Materials

Poly(lactic acid) (PLA 2003D, M_w_ = 236,000 g mol^−1^, 96 wt% L-isomer) was supplied by NatureWorks^®^ (Minnetonka, MN, USA) and poly(3-hydroxybutyrate) (PHB, M_w_ = 492,000 g mol^−1^) was provided by PHB Industrial S.A. (Serrana, SP, Brazil) under the name Biocycle^®^ L-61. Chitin from shrimp shells (Ch, practical grade, powder) and tributyrin (TB, 302 g mol^−1^, and 98.5% purity) were purchased from Sigma–Aldrich^®^ (Merck KGaA, Darmstadt, Germany). Hydrochloric acid (HCl, 37% *w*/*w*) was acquired from Anedra (Research Ag, Buenos Aires, Argentina).

### 2.2. Synthesis of Chitin Nanoparticles

Chitin nanoparticles (ChNPs) were synthesized from purified chitin powder by a hydrochloric acid hydrolysis following a process based on the method reported by Gopalan Nair et al. [36]. Hydrolysis was carried out with 3 M hydrochloric acid (HCl) at 90 °C for 90 min under vigorous stirring. The HCl to chitin powder ratio was of 30 mL g^-1^, aiming to hydrolyze the amorphous regions of the chitin and thereby decrease particle size [24,37]. After acid hydrolysis, cool, distilled water was added to stop the reaction and dilute the suspension five-fold. Further, the suspension was stored at 5 °C for 12 h and, after decantation, the supernatant was removed. Next, the suspension was transferred to dialysis bags and dialyzed in distilled water for 4 days until neutral pH was reached. The dispersion of nanoparticles was completed by a 5 min ultrasonic treatment for every 30 mL aliquot. The final suspension was freeze-dried in a VirTis 2KBTES-SS Lyophilizer (USA) to obtain dried powdered chitin nanoparticles.

### 2.3. Film Preparation

Prior to any processing, PLA, PHB, and ChNP were dried to avoid any moisture trace and undesirable hydrolysis reactions during the melt blending. Materials were dried overnight at 60 °C in a Cole-Parmer StableTemp vacuum oven (USA).

PLA/PHB blends were prepared by mixing PLA and PHB pellets in a double screw Haake mixer at 185 °C, a screw speed of 60 rpm, and a mixing time of 3 min. The weight ratio of PLA:PHB used was 60:40, and the proportion of plasticizer (TB) incorporated was 15 wt% of the final weight mixture, in accordance with previous works [33,34].

Plasticized PLA/PHB/TB blend was reinforced with 2 wt% of ChNP in order to manufacture the chitin nanocomposite, using a temperature of 185 °C and a screw speed of 60 rpm for a total time of 5 min. In this case, a suspension of ChNP and TB was used as feeding during the processing. The suspension was ultrasound-treated at room temperature for 1 h, stopping every 20 min to homogenize the mixture, in order to facilitate the dispersion of the nanoparticles in the plasticizer. Firstly, PLA pellets were put in the mixer until completely melted. Secondly, PHB pellets were incorporated. Finally, the ChNP-TB suspension was fed into the mixer after 3 min of blending PLA/PHB. The nanocomposite was mixed for two additional minutes. The process was performed in an inert atmosphere using nitrogen gas to avoid possible oxidation of the ChNP.

Blends were compression molded in an EMS AMS 160/335DE hydraulic press (Argentina) to obtain the films. The processing temperature was 190 °C and the pressure was kept 1 min at 0.1 MPa and 2 min at 5 MPa. Lastly, films were quenched at room temperature.

Neat and plasticized PLA and PHB films were also produced for comparison. The proportion of each component of the obtained binary, ternary, and quaternary formulations is summarized in Table 1.

### 2.4. Microscopy

The resulting ChNP morphology was characterized by field emission scanning electron microscopy (FE-SEM, Carl Zeiss NTS SUPRA 40, Oberkochen, Germany). A drop of highly diluted nanoparticles suspension was placed on a silicon plate and was left to dry. The sample was sputtered with a thin gold layer. The dimensions of the nanoparticles were obtained using SEM images and the ImageJ software, evaluating 250 representative items of the chitin nano-whiskers.

The microstructure of blends and bio-nanocomposite films’ cross-sections were observed by scanning electron microscopy (SEM, Jeol JSM-6460/LV, Beijing, China). The samples were previously frozen in liquid nitrogen, cryofractured, and then sputtered with a gold layer.

### 2.5. Infrared Spectroscopy

Fourier Transform Infrared spectroscopy analysis in attenuated total reflectance (FTIR-ATR) was performed using a Thermo Scientific (Nicolet 6700, Waltham, MA, USA) Instrument in the range of 4000–400 cm^−1^ by performing 32 overlapping scans at a resolution of 4 cm^−1^ at room temperature.

### 2.6. Wide Angle X-ray Scattering

Wide angle X-ray scattering (WAXS) measurements were carried out using a X’PertPro diffractometer (PANalytical, Almelo, The Netherlands) equipped with a fast solid-state X’Celerator detector, operating at 40 kV and 40 mA, with CuKα radiation (λ = 1.54 Å). Data were acquired in the 3–60° 2θ interval (acquisition time: 100 s; step: 0.10°).

### 2.7. Water Contact Angle Measurements

The surface hydrophobicity of films was studied by measuring the static water contact angle (WCA) by means of a KSV CAM101 goniometer (KSV Instruments, Inc., Helsinki, Finland) at ambient conditions by recording the side profiles of deionized water drops for image analysis. Ten drops were observed on different areas for each film, and contact angles were reported as the average value ± standard deviation. Each drop (4 uL) was deposited on the films by placing it in contact with the polymeric surface using a syringe needle (100 uL/min).

### 2.8. Thermal Analysis

Thermogravimetric analysis (TGA) was carried out using Perkin Elmer TGA7 (Waltham, MA, USA) apparatus under nitrogen atmosphere (gas flow: 40 mL/min) by heating from 30 to 700 °C at 10 °C/min. The specimen mass was in the range of 6–10 mg. The initial degradation temperatures (T_0_) were calculated at 5% mass loss, while thermal degradation temperatures at the maximum rate (T_max_) were determined from the first derivatives of the thermogravimetric curves (DTG).

Differential scanning calorimetry (DSC) was carried out on a Perkin Elmer DSC7 instrument (Waltham, MA, USA) under a nitrogen atmosphere (gas flow: 20 mL/min). The external block temperature control was set at −90 °C. The samples, of about 6 mg each, were encapsulated in aluminum pans and exposed to the following thermal treatment: (i) heating scan from −70 °C to 200 °C at 20 °C/min (first scan); (ii) isothermal scan at 200 °C for 1 min; (iii) cooling scan to −70 °C at 100 °C/min; (iv) isothermal scan at −70 °C for 14 min; (v) heating scan from −70 °C to 200 °C at 20 °C/min (second scan). The glass transition temperature (T_g_) was taken as the midpoint of the endothermic step associated to the glass-to-rubber transition. The melting temperature (T_m_) and the crystallization temperature (T_c_) were determined as the peak value of the endothermal and the exothermal phenomena in the DSC curve, respectively. The heat of fusion ($\Delta H_{m}$) and the heat of crystallization ($\Delta H_{c}$) of the crystalline phase were calculated from the total areas of the DSC endotherm and exotherm peaks, respectively. The degree of crystallinity ($\chi_{c}$) was calculated using Equation (1) [6].

(1) $$\chi_{c}=\left(\Delta H_{m}-\Delta H_{c}\right)/\left(\Delta H_{m}^{0}.\omega \right)\cdot 100\%$$

$\Delta H_{m}$ is the melting enthalpy, $\Delta H_{c}$ is the crystallization enthalpy, $\Delta H_{m}^{0}$ is the melting enthalpy of PLA or PHB 100% crystalline (93 J/g and 146 J/g, respectively) [38,39], and ω is the mass fraction of PLA or PHB in the sample.

### 2.9. Optical and Colorimetric Properties

The absorption spectra of film samples were obtained in the 190–1000 nm region using a UV–vis spectrophotometer (AGILENT 8453, Beijing, China). To convert absorbance values (A) to percent transmittance (%T), Equation (2) was used.

(2) A=2−log(%T)

Film color properties were evaluated by using the CIELab color space by means of a Lovibond Colorimeter RT500 (Amesbury, UK) with an 8 mm diameter measuring area, calibrated against a white standard tile. Color coordinates, $L^{*}$ (lightness), $a^{*}$ (green–red), and $b^{*}$ (blue–yellow) were measured along with percent opacity (%*Op*), and the average of three measurements at random positions over the film surfaces were reported. Total color difference (ΔE) was evaluated with respect to the white control according to Equation (3).

(3) $$\Delta E=\sqrt{{\left(\Delta L^{*}\right)}^{2}+{\left(\Delta a^{*}\right)}^{2}+{\left(\Delta b^{*}\right)}^{2}}$$

where $\Delta L^{*}=L^{*}-L_{0}^{*}$, $\Delta a^{*}=a^{*}-a_{0}^{*}$, and $\Delta b^{*}=b^{*}-b_{0}^{*}$; being $L_{0}^{*}$, $a_{0}^{*}$, and $b_{0}^{*}$ the color coordinates of the white standard.

### 2.10. Mechanical Properties

Uniaxial tensile tests were performed under ambient conditions using a universal testing machine (INSTRON 4467, Boston, MA, USA). Dumbbell shape specimens were investigated according to ASTM D1708-93, with a crosshead speed of 1 mm/min and a load cell of 500 N. Young’s modulus (E), tensile strength (σ_max_), and elongation at break (ε_b_) were calculated. Reported values were the average of at least five valid tests.

### 2.11. Barrier Properties

Water vapor permeability (WVP) of the films was evaluated following the methodology described by ASTM E96-00. Teflon capsules of 5 cm of diameter containing the film samples were placed in a chamber at 65% relative humidity and at an average temperature of 18 °C, using anhydrous calcium chloride (CaCl_2_) as a desiccant agent. The chamber allows controlling temperature and relative humidity with continuous air circulation to maintain uniform conditions at all test locations. Weight measurements were made using an analytical balance at regular time intervals until a steady state was reached. The WVP of the films was calculated according to Equation (4).

(4) WVP=WVTR.d/ΔP

*WVTR* is the water vapor transmission rate through the film (g/m^2^.s), *d* is the average film thickness (m), and Δ*P* is the difference in partial vapor pressure (Pa) between both sides of the film. Reported values were the average of three tests.

Oxygen (O_2_) and carbon dioxide (CO_2_) transmission rate was assessed by a manometric method using a Permeance Testing Device, type GDP-C (Brugger Feinmechanik GmbH, München, Germany), according to ASTM 1434-82 (Standard test Method for Determining Gas Permeability Characteristics of Plastic Film and Sheeting), DIN 53 536 in compliance with ISO/DIS 15 105-1 and according to Gas Permeability Testing Manual (Registergericht München HRB 77020, Brugger Feinmechanik GmbH). Method A was employed in the analysis, as reported in the Brugger manual, with the evacuation of top/bottom chambers. The film was placed between two chambers. The amount of gas flowing through the membrane was determined from the pressure variation due to the gas accumulation in the closed downstream chamber. All the measurements have been carried out at 23 °C. The operative conditions were a gas stream of 100 cm^3^·min^−1^; 0% RH of gas test, food grade; sample area of 0.785 cm^2^. The gas transmission rate (GTR) was determined considering the increase in pressure in relation to the time and the volume of the device. Gas transmission measurements were performed at least in triplicate and data was normalized for the thickness of the film samples.

### 2.12. Overall Migration in Food Simulants

Overall migration (OM) tests were performed according to the Commission Regulation EU No. 10/2011 on plastic materials and articles intended to come into contact with food [40]. A total migration test simulates the actual use of a plastic packaging material in contact with foodstuff and provides the total amount of non-volatile substances that could be transferred from the package to food. In addition, the European legislation establishes an overall migration limit (OML) of 10 mg dm^2^ (mass of migrant per dm^2^ of film) that should not be exceeded.

Two liquid food simulants were selected for analysis: 10% ethanol (*v*/*v*) (simulant A) and isooctane (alternative simulant to D1). Specifically, food simulant A is designated for food products that have a hydrophilic character; instead, simulant D1 is designed to simulate the behavior of food products that have a lipid character. Rectangular strips with a 25 cm^2^ total area of each film formulation were immersed in a glass tube with 25 mL of food simulant. Samples in 10% ethanol (*v*/*v*) were kept in a controlled chamber at 40 °C for 10 days, while samples in isooctane were kept at 20 °C for 2 days, according to EN-1186 standard. After the incubation period, the film samples were removed from the tubes, and the simulants were totally evaporated. Blank tests for each simulant under the same incubation conditions but without samples were also run. The non-volatile residue was determined by using an analytical balance. The overall migration (OM) values were expressed in mg dm^−2^ of film using Equation (5).

(5) $$OM=\left(M_{s}-M_{b}\right)/A_{s}$$

*M_s_* is the mass residue obtained after evaporating the simulant that has been in contact with the sample, *M_b_* is the mass residue of the blank test, and *A_s_* is the surface area of the sample.

All tests were performed in triplicate, and the overall migration was calculated as the average of these three determinations.

### 2.13. Disintegration under Composting Conditions

The study of the disintegration of the materials under aerobic composting conditions was carried out by a laboratory-scale test. Each film sample with dimensions 15 × 15 mm^2^ was placed in a 100 mL bottle and sandwiched between two layers of 20 g each of mature compost (kindly provided by HerAmbiente S.p.A., Bologna, Italy). Vessels were incubated in an SW22 Julabo water bath at 58 °C and 90 %RH.

Prior to degradation experiments, the samples’ dry weight was measured to obtain the sample initial mass. At different time intervals (3, 7, 14, 21, 28, and 35 days), duplicate sacrificial specimens of each sample were recovered, washed, and dried until the constant was weighed. Photographs of samples were taken for visual comparison. The disintegration degree (*D*) was determined gravimetrically according to Equation (6).

(6) $$D=\left(m_{i}-m_{r}\right)/m_{i}\cdot 100\%$$

*m_i_* is the initial weight of the dry sample mass and *m_r_* is the residual dry weight of the sample after the test.

## 3. Results and Discussion

### 3.1. Chitin Nanoparticles

**Figure 1:**
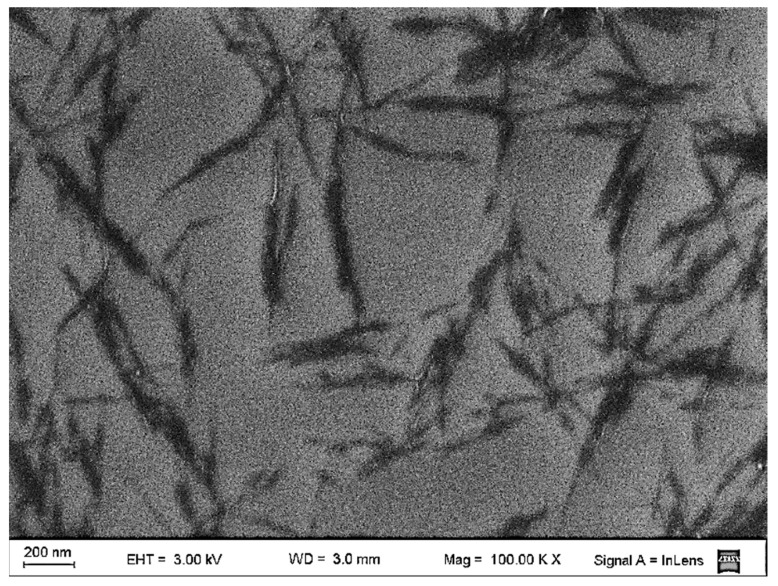
FE-SEM micrograph of dilute suspension of chitin nanoparticles in water.

Figure 1 shows the FE-SEM micrograph of the ChNPs obtained by hydrochloric acid hydrolysis. It was found that the chitin suspension was composed of individual and aggregated nanoparticles with a rod-like morphology that have a broad distribution size. The average dimensions of ChNP were obtained from the FE-SEM images. The average length (*L*), diameter (*d*), and its respective average aspect ratio (*L*/*d*) are summarized in Table 2. The dimensions of chitin nanoparticles found here are consistent with those obtained by other authors [36,37,41,42].

**Figure 2:**
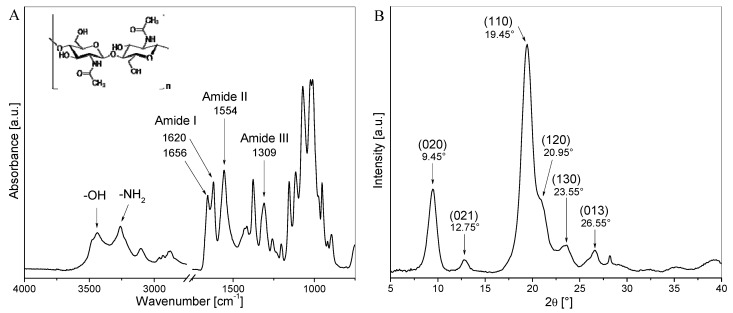
(**A**) FTIR spectrum and (**B**) WAXS patterns of dry powdered chitin nanoparticles.

The FTIR-ATR spectrum of dry ChNP powder is shown in Figure 2A. The chitin nanoparticles presented characteristic peaks of pure α-chitin: the absorptions centered at about 3400 cm^−1^ are attributed to the −OH and −NH_2_ groups’ stretching vibration and intermolecular hydrogen bonding; the bands at 1656 and 1620 cm^−1^ correspond to the amide I region (stretching of the C=O group of the peptide bonds); the peak at 1554 cm^−1^ corresponds to the amide II band (N-H bending); and the peak at 1309 cm^−1^ is attributed to the amide III vibration (C-N stretching) [43]. All the mentioned bands are indicated in Figure 2A, in which the chemical structure of chitin was also included for a better understanding.

The crystalline structure of ChNP was studied by WAXS (Figure 2B). The diffraction pattern of the chitin nanocrystals shows the typical reflections of pure α-chitin, indicating that the crystal integrity is maintained after hydrolysis [37]. The six most intense crystalline diffraction peaks were observed at 2θ values of 9.45°, 12.75°, 19.45°, 20.95°, 23.55°, and 26.55°, and were indexed as the reflections for the crystalline planes (020), (021), (110), (120), (130), and (013), respectively, according to the orthorhombic structure of α-chitin [37,44].

**Figure 3:**
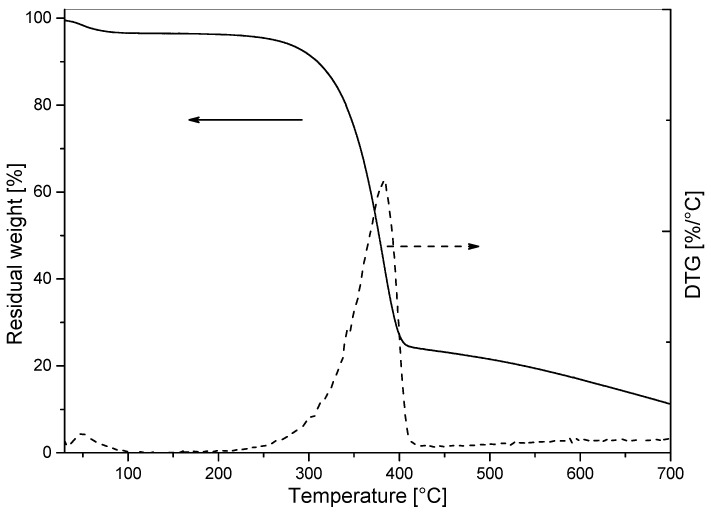
TG and DTG thermograms of dry powdered chitin nanoparticles.

TGA assays were carried out to investigate the thermal stability and degradation profile of chitin nanocrystals. Figure 3 shows the residual weight vs. temperature curve (TG) and its corresponding derivative (DTG). Thermal degradation of ChNP occurred in a single degradation process under a nitrogen atmosphere, with an initial degradation temperature (at 5% mass loss) of 260 °C and a maximum degradation temperature at 385 °C, leaving a mass residue of nearly 11% at 700 °C. Therefore, the nanocrystals are thermally stable at the processing temperature range, which was below 200 °C.

### 3.2. PLA/PHB Bio-Nanocomposite

#### 3.2.1. Morphological and Structural Characterization

**Figure 4:**
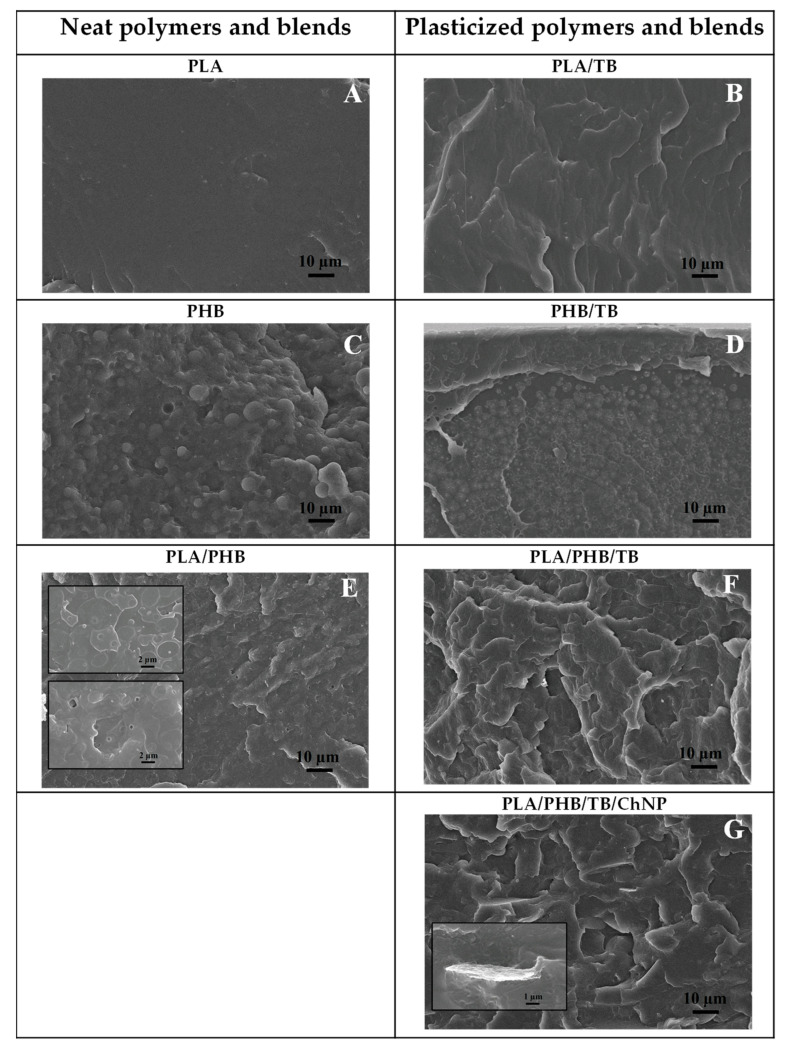
SEM micrographs of fracture surface of (**A**) PLA, (**B**) PLA/TB, (**C**) PHB, (**D**) PHB/TB, (**E**) PLA/PHB, (**F**) PLA/PHB/TB, and (**G**) PLA/PHB/TB/ChNP.

Figure 4 shows the SEM micrographs of the cross-fractured sections of the different materials’ films. Pure PLA (Figure 4A) exhibited a smooth and uniform fracture surface characteristic of an amorphous brittle polymer, while pure PHB (Figure 4C) showed an irregular fracture surface due to its crystalline structure. Plasticized PLA and PHB fracture surfaces (Figure 4B,D) showed more plastic deformation than pure polymers did and no apparent phase separation because of the homogeneous dispersion of TB in the polymeric matrices confirming the efficiency of TB as a plasticizer. PLA/PHB film (Figure 4E) presented a rough fracture surface and revealed two types of microstructures (magnified in the inset picture), which would suggest phase separation between PLA and PHB. In addition, the presence of some voids of different sizes were detected (highlighted by white arrows in Figure 4E and magnified in the inset picture); this would indicate the debonding of the dispersed PHB particles from the PLA matrix. The immiscible and/or partially miscible nature between PLA and PHB phases has also been determined in our previous studies [5,33]. The incorporation of TB in the PLA/PHB formulation (i.e., PLA/PHB/TB ternary system, Figure 4F) caused the disappearance of those voids and smoothed the surface morphology, showing more fuzzy interfaces and, consequently, a better adhesion between the PLA matrix and the PHB inclusions. The PLA/PHB/TB/ChNP nanocomposite (Figure 4G) exhibited the distribution of some compact structures (pointed out by white arrows in Figure 4G), suggesting that the ChNPs are present as micro-sized agglomerates with a flake shape (magnified in the inset picture). This could be explained by the formation of hydrogen bonds between the nanocrystals and their tendency to agglomerate during the blending process [45].

**Figure 5:**
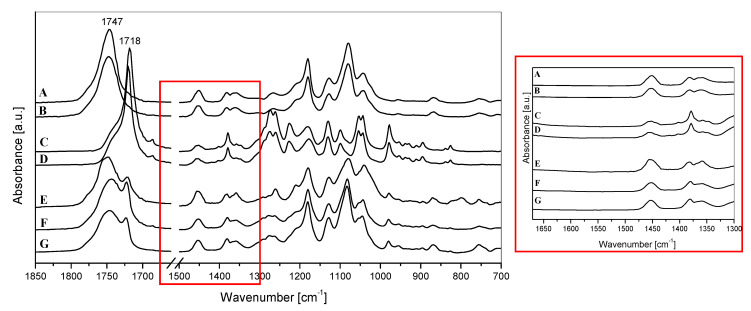
FTIR spectra in the 1850–700 cm^−1^ region of (**A**) PLA, (**B**) PLA/TB, (**C**) PHB, (**D**) PHB/TB, (**E**) PLA/PHB, (**F**) PLA/PHB/TB, and (**G**) PLA/PHB/TB/ChNP. Inset: FTIR spectra in the 1670–1300 cm^−1^ region.

The FTIR spectra of PLA- and PHB-based materials are shown in Figure 5 in the 1850–700 cm^−1^ region. The characteristic peaks corresponding to the asymmetric stretching of the carbonyl group (C=O) in the polyesters were observed at 1747 and 1718 cm^−1^ for pure PLA and PHB, respectively [46]. As expected, all spectra of PLA/PHB-based blends displayed the two major carbonyl stretching bands, with one due to PLA and the other to PHB, respectively. No apparent changes were observed in the spectrum of the PLA/PHB/TB/ChNP nanocomposite with respect to the PLA/PHB/TB sample. Moreover, the chitin characteristic absorption bands corresponding to the amide groups were not detected in the spectrum of nanocomposite, which is probably attributable to the low content of ChNP used (see the inset of Figure 5).

**Figure 6:**
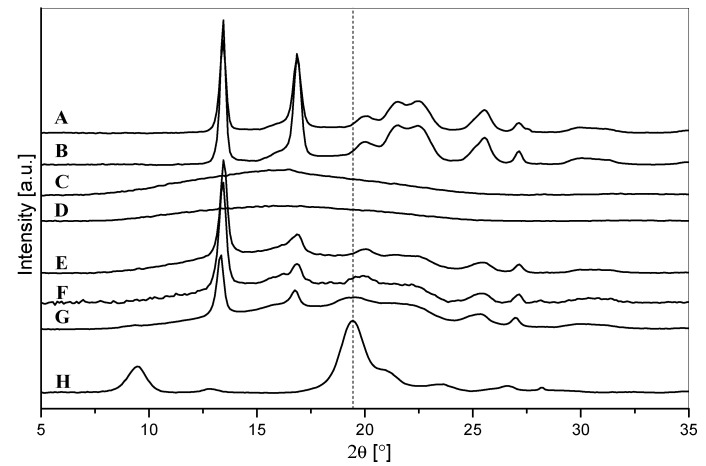
X-ray diffraction patterns of (**A**) PHB, (**B**) PHB/TB, (**C**) PLA, (**D**) PLA/TB, (**E**) PLA/PHB, (**F**) PLA/PHB/TB, (**G**) PLA/PHB/TB/ChNP, and (**H**) ChNPs.

In order to investigate the crystalline structure of the developed materials, X-ray diffraction tests were performed. The diffractograms are shown in Figure 6. Both PHB and PLA are known to be able to crystallize from the molten state in an orthorhombic unit cell [6]. Pure processed PHB was found to be highly crystalline, showing two strong diffraction peaks located at 2θ = 13.5 and 16.9°, associated with (020) and (110) planes, respectively, and six weaker peaks at 2θ = 19.9, 21.5, 22.4, 25.5, 27.1, and 30.4° (Figure 6A). These peaks are assigned to the (021), (101), (111), (121), (040), and (002) planes, respectively [47]. On the other hand, pure processed PLA displayed a single-wide diffraction band typical of an amorphous polymer, located at 2θ between 10 and 25° (Figure 6C) [48]. The patterns of PHB/TB and PLA/TB (Figure 6B,D) were almost identical to that of pure polymers.

In general, the diffraction patterns of all the PLA/PHB blends (Figure 6E–G) were very similar to that of PHB; however, the intensities of the peaks were weaker, hidden by the amorphous halo characteristic of the PLA matrix. Accordingly, the peak located at 16.9° and associated with the (110) PHB plane was significantly weaker because it coincides with the center of the amorphous band. Similar results were found by Zhang et al. [48] when they studied PLA/PHB blends in a number of different weight ratios.

The presence of ChNP in a PLA/PHB/TB/ChNP nanocomposite (Figure 6G) was evidenced by the slight widening of the left-hand shoulder of the (021) PHB reflection (2θ = 19.9°), exactly where the strongest (110) ChNP reflection (Figure 6H) is positioned (2θ = 19.45°).

#### 3.2.2. Water Contact Angle

**Figure 7:**
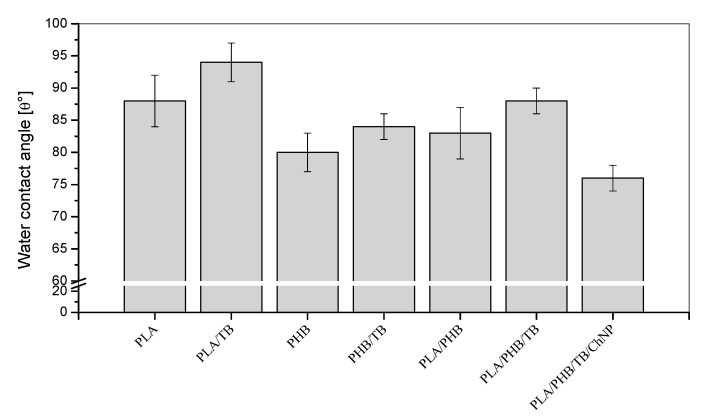
Contact angle measurements of PLA- and PHB-based materials.

Since materials intended for food packaging are required to protect foodstuff from humidity, the wettability of films was evaluated. The hydrophilic/hydrophobic behavior of the surface film samples was investigated by static water contact angle (θ) measurements. Results are shown in Figure 7. In general, the prevailing definition of the limit between hydrophilic and hydrophobic surfaces is a contact angle smaller or larger than 90°, respectively. However, Vogler’s research has shown that this limit should be reduced to around 65° [49]. Based on this, all studied materials showed values higher than 65° and, thus, turned out to be hydrophobic, showing their surfaces’ poor affinity of water. This characteristic is favorable for materials planned to be subjected to conditions of high relative humidity, such as packaging applications.

The contact angles for PLA/TB, PHB/TB and PLA/PHB/TB films were around 6% higher than their un-plasticized counterparts, suggesting that the addition of 15% of TB produced a reduction in materials’ surfaces wettability. Similar results were observed in PLA-PHB matrices plasticized with acetyl tributyl citrate (ATBC) or D-limonene [4,50], and this behavior is attributed to the hydrophobic nature of those additives. The presence of ChNP in the nanocomposite caused a 14% reduction in θ compared with the PLA/PHB/TB film, probably due to the incorporation of chitin hydroxyl groups into the films [21].

#### 3.2.3. Optical and Colorimetric Properties

**Figure 8:**
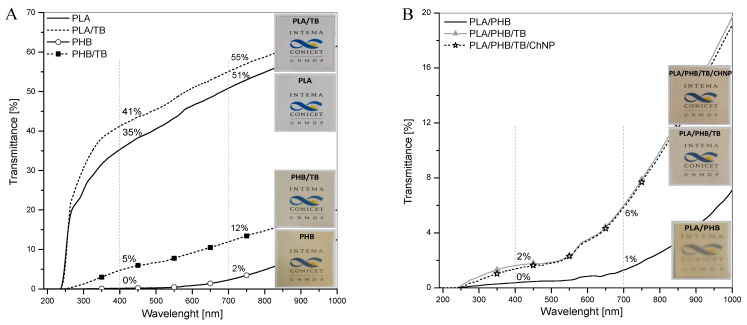
UV–vis spectra and visual appearance of prepared films (**A**) PLA, PLA/TB, PHB, PHB/TB, and (**B**) PLA/PHB, PLA/PHB/TB, PLA/PHB/TB/ChNP.

Color and optical properties are of importance for materials intended for food packaging purposes. Figure 8 shows the visual appearance of developed films and their UV–vis transmittance spectra, while Table 3 summarizes the color parameters and percent of opacity of the different formulations.

The transmission of UV and visible light through polymers is one of the main factors affecting food quality. For instance, light has been found to affect the flavor and the nutritional content of some products [51]. In this context, the primary wavelengths (λ) of interest in packaging applications are those that fall between 100 and 700 nm. This section of the electromagnetic spectrum can be divided into two components: ultraviolet (UV) band (100–400 nm) and visible spectrum (400–700 nm). UV radiation is subdivided into three distinct wavelength regions. UV-A (400–315 nm) is the longest wavelength region and lowest in energy; UV-B (315–280 nm) is the most energetic component of natural UV light and causes the most photochemical degradation of plastics; UV-C (280–100 nm) is generally created from artificial light sources [52].

Neat PLA sample (Figure 8A) showed no UV transmission in the lower range of UV-C (190–240 nm); however, at longer wavelengths, the percent transmittance increased significantly, reaching the 35% at λ = 400 nm. Hence, a large amount of UV-B and UV-A light passes through the film. Moreover, PLA film showed high transparency, since it exhibited a high transmittance percentage in the entire visible light band region (51%T at λ = 700 nm). On the other hand, the neat PHB sample (Figure 8A) presented null values over the entire UV region, giving evidence of its good UV barrier properties in comparison to PLA [53], and it showed the lowest transmittance value at 700 nm (2%) among the films studied, which is directly related with low transparency. The plasticization of both polyesters increased the light transmittance throughout the whole wavelength region measured. The PLA/PHB sample (Figure 8B) exhibited a light barrier behavior almost identical to that of pure PHB. After the addition of TB into the PLA/PHB blend, the transmittance of visible light was increased (6%T at λ = 700 nm) while maintaining excellent UV barrier properties. Finally, the incorporation of ChNP did not modify the transparency of the PLA/PHB/TB films.

Despite of the different transmittance percentages obtained within the visible light range (400–700 nm), all developed films showed good transparency since the logo situated under them in the pictures of Figure 8 can be clearly seen. Each wavelength in the visible light band causes a particular sensation of color [52]. One of the most common systems used to characterize colorimetric properties is the CIELab color space, which is used to determine and to compare the color of the samples.

Neat PLA showed the highest $L^{*}$ value representative of its high brightness, $a^{*}$ and $b^{*}$ values close to zero are consistent with its colorless nature, and the lowest %*Op* is indicative of its high transparency (Table 3). Similar results were obtained for the PLA/TB film. Both materials displayed values of Δ*E* equal to or less than 2, which indicate that the total color differences of the films relative to the white control were below the Just Noticeable Difference threshold by the human eye [54]. Lightness values of the PHB and PHB/TB samples were lower than that of the PLA and PLA/TB films, and the increment of the parameters $a^{*}$ and $b^{*}$ are indicative of a red-yellowish coloration, which is due to the typical amber color of PHB. This tendency was also reflected in the significant value of the total color difference (Δ*E*). Additionally, the PHB sample presented a %*Op* two-fold higher than pure PLA due to its crystalline nature. The PLA/PHB sample turned out to be the least transparent among the materials studied with an 18%*Op*. Opacity in immiscible polymer blends can be originated from different refractive indexes between the two domains and/or due to the scattering of light due to interfacial voids [55]. Its $L^{*}$ value did not significantly change with respect to the neat PLA; however, the PHB presence produced a stronger tendency towards yellow, showing an increment in $b^{*}$ coordinate. PLA/PHB/TB formulation showed a decrease in %*Op*, indicating an improvement in transparency compared with the PLA/PHB blend. The subsequent incorporation of ChNP caused a slight increase in the $b^{*}$ parameter and in Δ*E* for the PLA/PHB/TB/ChNP sample—a possible result of the typical yellowish coloration of chitin.

These results indicate that the PLA/PHB/TB and PLA/PHB/TB/ChNP nanocomposites have better UV barrier properties than PLA or PLA/TB, demonstrating their potential applicability as fully biomass packaging and/or coating systems characterized by transparency and exceptional UV-protection capability, which are mainly significant for light-sensitive products [56].

#### 3.2.4. Thermal Characterization

**Figure 9:**
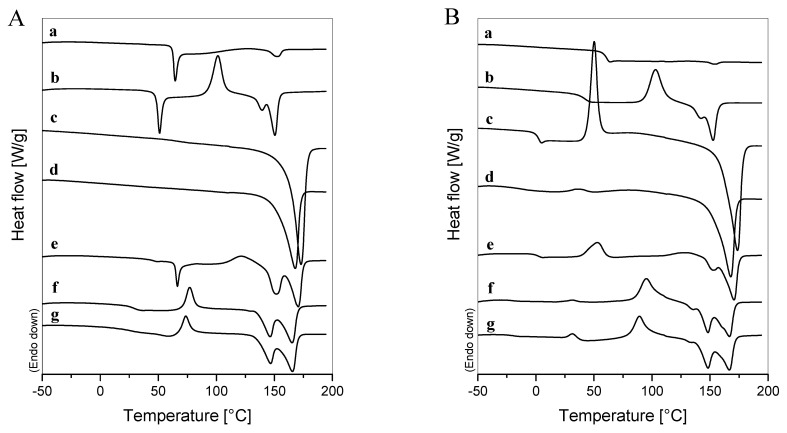

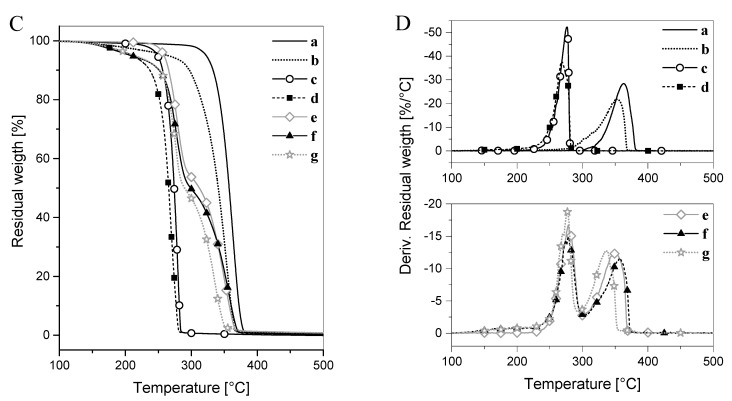
Thermal analysis results of (a) PLA, (b) PLA/TB, (c) PHB, (d) PHB/TB, (e) PLA/PHB, (f) PLA/PHB/TB, and (g) PLA/PHB/TB/ChNP: (**A**) DSC first and (**B**) second heating scans; (**C**) TG and (**D**) DTG thermograms.

Differential scanning calorimetry was carried out to investigate the film samples’ thermal characteristics and the role of ChNP on bio-nanocomposite thermal behavior. Figure 9A,B shows the DSC first and second heating scans of PLA- and PHB-based materials, and Table 4 summarizes the acquired data from the first scan.

The T_g_ value for the neat PLA sample was found at 62 °C, while weak peaks corresponding to the exothermic crystallization and endothermic melting of PLA were observed at around 124 and 152 °C, respectively. The addition of plasticizer induced a significant depression in T_g_ and T_c_ values and a slight reduction in T_m_ for the PLA/TB film sample due to the higher molecular mobility of the polymer chains, confirming the efficiency of TB as a plasticizer. Moreover, the presence of TB increased the ability of PLA to crystallize, revealed not only by the shift of T_c_ to lower temperatures, but also by an exothermic crystallization enthalpy value ten times higher than the one of the neat PLA (Table 4). The PLA crystallization enhancement due to plasticization has already been reported by many authors [57,58,59]. Nonetheless, the total enthalpy values of the continuous transitions that occurred in the range between 90 and 160 °C ($\Delta H_{m}-\Delta H_{c}$) during the first heating scans for PLA and PLA/TB were close to zero, indicating the amorphous nature of both processed films quenched at room temperature. X-ray diffraction patterns of neat and plasticized PLA samples (Figure 6C,D) confirmed their amorphous state after processing. Burgos et al. [60] found similar results by studying the crystallinity of PLA films plasticized with different concentrations of oligomeric lactic acid. A double melting peak behavior was found for the PLA/TB sample (Figure 9A,B) that could be attributed to the melting of two different kinds of crystal structures. Zhang et al. [61] reported the existence of a relationship between the crystal modifications of PLA and the crystallization temperature. When T_c_ is lower than 100 °C, disordered α’-crystals with low thermodynamic stability are formed, while order α-crystals are obtained at T_c_ above 120 °C. At crystallization temperatures between 100 and 120 °C, it was proposed that PLA crystallizes into both crystal modifications (α’ and α), presenting a double melting peak behavior [60,61], as it was in the case of PLA/TB.

The first DSC heating scan for neat PHB exhibited one strong endothermic melting peak at around 173 °C (Figure 9A), characteristic of its crystalline nature. The incorporation of TB reduced the T_m_ value in the PHB/TB sample [62,63]. On the second heating scans (Figure 9B), the T_g_ and T_c_ of PHB at around 1 and 50 °C, respectively, could be detected, as could the subsequent depression of these temperatures in the PHB/TB sample due to plasticization. Therefore, it can be inferred that TB is completely miscible and effective as plasticizer for both PLA and PHB, as has already been shown in our previous works [5,33]. Regarding the degree of crystallization, the additive caused a small decrease in the χ_c_ values of PHB/TB in respect to neat PHB (Table 4). The PHB microcrystals or ordered chains tend to have greater mobility with the plasticizer content and could be more easily moved to pack into a less dense or unperfected crystalline structure [62].

The PLA/PHB blend showed a double glass transition behavior, one for each polyester (T_g,PHB_ was only detected on the second heating scan), and multi-step melting, with the first peak being due to PLA and the second one corresponding to PHB. Moreover, the DSC curves displayed no considerable variations of the thermal characteristic properties with respect to individual neat polymers (Table 4), suggesting the immiscibility between them [64], as aforementioned in the morphological characterization. In addition, it is interesting to see that the thermograms of PLA, PLA/TB, and PLA/PHB (a,b,e in Figure 9A) revealed a sharp endothermic peak superimposed on the heat flow shift associated with the glass transition of PLA. This is attributed to the enthalpy of relaxation due to a possible physical aging of the polymer during the storage at room temperature, which is usually observed in amorphous polymers [65].

A double melting behavior also occurred for PLA/PHB/TB and PLA/PHB/TB/ChNP blends (f,g in Figure 9A), indicating phase separation. However, it was possible to note that the presence of TB lowered the T_g_, T_c_, and T_m_ values of PLA in these samples even more than in that of PLA/TB. For instance, the T_g,PLA_ located at about 46 °C in the PLA/TB sample was shifted to 29 °C, with the incorporation of PHB in the PLA/PHB/TB sample. This could be due to partial miscibility between PLA and PHB when they are plasticized with TB, confirming its efficiency as a plasticizer and compatibilizer [33,66,67]. Moreover, the T_m_ value of PHB in PLA/PHB/TB and PLA/PHB/TB/ChNP blends was almost 10 °C lower than that of pure PHB. This result shows that the blends could be melt processed and compression molded at lower temperatures, improving the narrow processing window of PHB which usually presents thermal degradation by random chain scission when the processing temperature reaches 190 °C [63]. This result was also corroborated by the TGA analysis shown below.

The crystallinity degree of the polymers in PLA/PHB blends and the nanocomposite was calculated using the thermal enthalpies acquired from the first heating curves (Table 4) and Equation (1). Endothermic melting peaks corresponding to the melting of each polymer were obtained by mathematical deconvolution using a Gaussian multi-peak fit on the software OriginPro 8.5. The PHB dispersed crystal phases acted as nucleating centers and induced a considerably increase in the χ_c_ of the PLA in the PLA/PHB sample [48], which was then slightly reduced in the PLA/PHB/TB sample, caused by the subsequent plasticization with TB [15]. Finally, the thermal results for the PLA/PHB/TB/ChNP nanocomposite revealed a slight reduction in T_c_ for PLA compared with the unfilled PLA/PHB/TB sample, indicating that the PLA’s ability to crystallize was enhanced. Moreover, χ_c,PLA_ was higher in the filled film sample due to the nucleation effect attributed to the dispersed ChNP. The capacity of chitin nanocrystals to act as a nucleating agent in PLA-based nanocomposites has already been reported by Herrera et al. [27]. PHB crystallinity was found to be lower in PLA/PHB blends with respect to neat PHB, probably due to the limited conformational mobility of its chains restricted by the glassy regions of PLA. However, the addition of ChNP increased the χ_c,PHB_ compared to the plasticized blend, as was observed for PLA.

Thermogravimetric analysis of processed materials was also conducted. The TG and DTG curves and the main thermal parameters obtained from them are shown in Figure 9C,D and Table 4, respectively. Neat PLA and neat PHB degraded in one-step processes. As expected, PHB was less thermally stable than PLA, presenting its maximum degradation rate centered at 277 °C. The plasticizer presence reduced the initial degradation temperature (T_0_) of both polymers in the PLA/TB and PHB/TB samples, respectively, in good agreement with other plasticized PLA [60] and plasticized PHB systems [63].

PLA/PHB, PLA/PHB/TB, and PLA/PHB/TB/ChNP materials degraded in two-step processes. Once again, the initial thermal stability of the blend was compromised by plasticization but the addition of highly stable ChNP slightly shifted the T_0_ to upper temperatures. It should be noted that no degradation takes place within the temperature range from ambient temperature to 200 °C, where the biodegradable nanocomposite and blends are processed and intended to be used.

#### 3.2.5. Mechanical Characterization

Mechanical properties of PLA/PHB-based materials plasticized with TB have been extensively studied in our previous published works [33,34]. In order to investigate the role of ChNPs on the bio-nanocomposite tensile behavior, new mechanical tensile tests were performed, and the results are summarized in Table 5.

The PLA/PHB sample showed the comparable mechanical properties’ values to the neat polymers’ films. As expected, the TB plasticization of PLA, PHB, and PLA/PHB films reduced the Young’s modulus and tensile strength of PLA/TB, PHB/TB, and PLA/PHB/TB films. However, while it did not enhance the elongation at the break performance of the first two compositions, the addition of TB to the binary polymer blend caused a significant increase in flexibility. The plasticized PHB phase also dispersed into a plasticized PLA matrix, which would induce deformation mechanisms which would improve the ductility of the blends [5,68]. This is in good agreement with DSC results, where the PLA/PHB/TB film displayed a lower T_g_ value than the plasticized PLA counterpart (Table 4). On the other hand, the incorporation of ChNPs into the last-mentioned system proved to be effective in increasing the *E* value; however, the material showed a brittle failure. In general, the elongation at break in reinforced polymers is affected by the volume fraction of the added filler, its dispersion in the matrix, and its interaction with the polymer matrix [69]. In this particular case, chitin nanocrystals were somewhat agglomerated and caused substantial local stress concentrations, provoking failure at low strain values. In addition, the difference of components’ surface polarity was not advantageous to obtain a good interfacial adhesion between PLA, PHB, and ChNP. Similar behaviors have been reported by other authors when they observed that the addition of chitin or cellulose nanocrystals into a PLA or PLA/PHB matrix resulted in the drastic decrease in elongation for the break values of nanocomposites [15,28,69,70].

#### 3.2.6. Barrier Properties

The barrier properties are considered important parameters in food packaging manufacturing due to the role of water vapor and various gas transmission in deteriorative reactions and microbial and mold growth [71]. Hence, water vapor permeability (WVP), as well as oxygen (O_2_) and carbon dioxide (CO_2_) transmission rates through the films, were evaluated, and the results are shown in Table 5.

Neat PHB-processed film presented greater resistance to the transmission of dry gases and water vapor due to its crystalline nature. The gas and vapor permeability of semi-crystalline polymers is mainly affected by the percentage of a crystalline phase which is impermeable to gases, therefore a higher χ_c_ makes the polymer less permeable [72]. The incorporation of 40 wt% of PHB to the PLA matrix greatly enhanced the barrier properties of the latter, as can be seen in the reduced values of GTR and WVP for the PLA/PHB sample in respect to neat PLA.

Plasticization of PLA, PHB, and PLA/PHB samples increased the gas and vapor transmission through the PLA/TB, PHB/TB, and PLA/PHB/TB films as a result of the increase in free volume and chain mobility [33,62,73,74]. It is interesting to note that, even though PHB/TB was the most crystalline sample, the permeability competence was the poorest one within this series. The incorporation of flexible segments of TB into a PHB dense and crystalline matrix would weaken the interactions between the ordered chains of PHB and conform to a much less dense structure [62,75], as mentioned before in the thermal characterization section. On the other hand, neat PLA is essentially amorphous, therefore its plasticization would favor the steric accommodation of polymer chains, causing a more ordered structure and, thus, a less pronounced increase in permeability. Finally, the further incorporation of chitin nanoparticles did not significantly affect the water vapor permeability of the PLA/PHB/TB/ChNP nanocomposite [27], but did reduce its gas transmission rate of dry oxygen and carbon dioxide compared with the PLA/PHB/TB sample.

For all samples under consideration, CO_2_ was the gas that propagated fastest through the films, despite its larger dimension. Similar results were found by some of us when studying the highly hydrophobic polymer matrix [76]. It should be highlighted that the O_2_ and CO_2_ transmission rate values for the PLA/PHB/TB blend and the PLA/PHB/TB/ChNP nanocomposite are clearly lower than those of commercial low-density polyethylene (LDPE), namely 19.5 and 78 cm^3^ cm m^−2^ d^−1^ atm^−1^, respectively [76], therefore they could be acceptable in these terms for food packaging.

#### 3.2.7. Overall Migration

Overall migration (OM) tests were carried out on PLA/PHB bio-nanocomposite films using isooctane and 10% ethanol (*v*/*v*) as food simulants. Table 5 shows the OM values of neat and plasticized PLA-, PHB-, and ChNP-based formulations in both polar and non-polar simulants.

In the case of isooctane (non-polar), all materials showed overall migration values lower than the current limit (10 mg dm^−2^). Yet, it was observed that the migration levels increased when samples were plasticized with TB. On the other hand, migration tests performed in the polar simulant showed that PLA/TB, PHB/TB, and PLA/PHB/TB formulations exceeded the OML. This behavior could be related to the plasticizing effect of TB, resulting in an increase in free volume and the mobility of polymer chains within the matrix, as was previously reported in our preceding work [35] and as in agreement with the decrease in the glass transition temperatures of polymers determined by DSC (Table 4).

The PLA/PHB/TB/ChNP nanocomposite was detected to have reductions of 18 and 34% in the migration values in isooctane and 10% ethanol (*v*/*v*), respectively, compared to the unfilled counterpart blend (PLA/PHB/TB film). Then, the incorporation of 2 wt% of ChNP into the polymer matrix shifted the OM values in the 10% ethanol (*v*/*v*) simulant to a value below the OML. The interaction between the ChNPs and the polymeric matrix could have caused a restriction in the polymer chains’ mobility, lowering the migration [77].

The migration results underlined the positive effect of the incorporation of the low amount of chitin nanoparticles that could act as nucleating agents, increasing the crystallinity degree, as shown by the DSC thermal characterization, and thus enhancing the migration performance.

#### 3.2.8. Disintegration under Composting Conditions

Biodegradability, specifically disintegration under composting conditions, is one of the most attractive properties of biopolymers intended for packaging applications in order to limit the serious problem of waste disposal [78]. Biodegradation of the PLA/PHB blends plasticized with TB have been studied in detail in our previous article [35]. In this work, the effect of the addition of chitin nanoparticles on the biodegradation of plasticized PLA/PHB blends was investigated.

**Figure 10:**
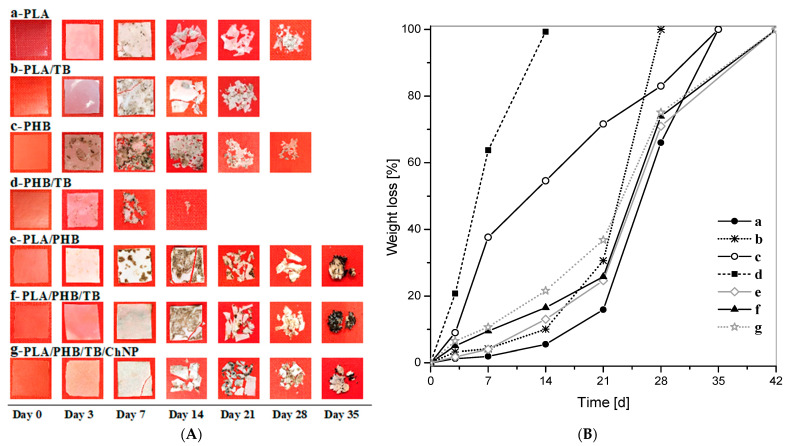
Disintegration under composting conditions before and after different stages of incubation of (a) PLA, (b) PLA/TB, (c) PHB, (d) PHB/TB, (e) PLA/PHB, (f) PLA/PHB/TB, and (g) PLA/PHB/TB/ChNP: (**A**) visual observation and (**B**) disintegrability percentage values.

Figure 10A,B show photographs of the materials’ samples and their disintegration percentage evolution at different incubation times, respectively. All films changed their color and opacity just after 3 days of incubation in composting conditions as a consequence of increased crystallinity and the beginning of the hydrolytic degradation process [13,79], while extensively noticeable fractures appeared after 14 days (Figure 10A).

Neat PHB degraded with a higher disintegration rate than neat PLA, reaching a mass loss of 38% after 7 days, while PLA only achieved a disintegration percentage of 2% under the same conditions and incubation time (Figure 10B). It is known that the PHB degradation is mainly caused by polymer surface erosion produced by microorganisms, which then are able to propagate gradually to the interior of the polymer matrix [80]. Thus, PHB mass loss was registered from the start of the test. Meanwhile, the PLA degradation begins with a non-enzymatic hydrolysis process leading to the random chain scission of the ester groups of the polymer backbone [81]. This almost two-week first stage, in which the molecular weight decreases but the residual gravimetric weight remains nearly constant [82], is followed by the metabolization of the low molecular weight hydrolysis products by microorganisms to yield carbon dioxide and water [83]. Accordingly, the degradation of neat PLA began slowly and, after the third week, sped up.

The plasticization of pure polymers resulted in a significant acceleration of the biodegradation rate for PLA/TB and PHB/TB. The small TB molecules are more susceptible for bacteria and fungi attack and also increase polymer chain mobility [4,70], as confirmed by the depression in T_g_ values (Table 4).

The progressive addition of the different components (PHB, TB, and ChNP) into the PLA matrix improved its degradation kinetics, being the observed trend PLA < PLA/PHB < PLA/PHB/TB < PLA/PHB/TB/ChNP. Chitin nanoparticles (ChNPs) sped up the disintegration rate of the PLA/PHB/TB blend mainly during the first 3 weeks of incubation. As aforementioned, the disintegration in the compost of the PLA matrix starts by a hydrolysis process [81]; thus, the accelerated disintegration process could be ascribed to the more hydrophilic character of ChNP, which would make the polymer matrix and film surface more vulnerable to the water attack [14]. This is in good agreement with the lower WCA value displayed by the nanocomposite (Figure 7). The chitin hydroxyl groups available on the film surface would catalyze the hydrolysis of the polymer chains, leading to a higher disintegration rate for PLA/PHB/TB/ChNP compared to PLA/PHB/TB. Similar biodegradation rate tendencies were observed by other authors when studying the compostability of PLA, PHB, and/or PLA/PHB nanocomposites with cellulose nanocrystals [14,84,85].

In brief, all formulations were fully disintegrated under composting conditions within one and a half months, suggesting their respective advantages in industrial applications when short biodegradation times are required.

## 4. Conclusions

Plasticized nanocomposite films based on PLA/PHB/TB reinforced with synthesized ChNP were developed and deeply characterized. Chitin nanoparticles were successfully obtained by hydrochloric acid hydrolysis.

The resulting nanocomposite film was optically transparent to visible light and opaque in the UV region, which is particularly advantageous for packaging light-sensitive products. The presence of ChNP on the surface of the specimens was detected by a reduction in the WCA measurements. The addition of ChNP to the PLA/PHB/TB blend contributed to a reduction in the melting temperature of PHB as well as to an increment in the initial thermal degradation temperature of the film, thus leading to an improvement in the typically narrow processing window of the neat PHB. The synergic effect of PHB and ChNP enhanced the crystallization of the PLA matrix and improved both the gas barrier properties and the overall migration behavior of plasticized PLA/PHB blends. The composting test confirmed the biodegradable character of all film formulations. Moreover, the tributyrin and chitin nanoparticles were able to speed up the disintegration process of the produced materials. The tensile properties of the films showed a reinforcing effect of chitin nanoparticles by increasing their rigidity and strength, while their elongation was greatly reduced. The absence of a ductile behavior was explained by the poor interfacial adhesion between the components that would be insufficient to withstand interfacial stresses generated during tensile deformation. An adequate amount of filler and/or a modification in nanoparticles’ surface polarity would enhance the interfacial adhesion and thus the mechanical properties.

These results suggest that chitin nanoparticles are promising filler for the preparation of multifunctional materials based on plasticized PLA/PHB blends and that may offer good perspectives for food packaging applications. Further investigation is currently in progress to modify chitin whiskers’ surfaces, leading to higher hydrophobicity in order to increase their dispersion in the non-polar polymer matrix to promote interfacial adhesion and to enhance the final properties of nanocomposite systems while taking into account the proposed field of application.

## Figures and Tables

**Table 1:** Compositions of the obtained materials.

	PLA [wt%]	PHB [wt%]	TB [wt%]	ChNP [wt%]
PLA	100	/	/	/
PLA/TB	85	/	15	/
PHB	/	100	/	/
PHB/TB	/	85	15	/
PLA/PHB	60	40	/	/
PLA/PHB/TB	51	35	15	/
PLA/PHB/TB/ChNP	49.8	33.2	15	2

**Table 2:** Dimensional properties of chitin nanoparticles (values in brackets correspond to the standard deviation).

Average Length, *L* [nm]	Average Diameter, *d* [nm]	Aspect Ratio, *L*:*d*
300 (170)	40 (10)	7.5:1

**Table 3:** Thickness, color parameters ($L^{*}$: lightness, $a^{*}$: green–red, $b^{*}$: blue–yellow), and opacity for PLA- and PHB-based materials (values in brackets correspond to the standard deviation).

	Thickness [μm]	$L^{*}$	$a^{*}$	$b^{*}$	ΔE	*Op* [%]
White Control		92.28 (0.00)	1.38 (0.00)	−0.86 (0.00)		
PLA	118 (5)	94.21 (0.08)	−1.15 (0.01)	0.11 (0.03)	2.09 (0.09)	8.5 (0.1)
PLA/TB	119 (4)	93.37 (0.30)	−1.18 (0.02)	0.49 (0.09)	1.16 (0.30)	8.4 (0.5)
PHB	108 (2)	85.14 (1.24)	0.47 (0.25)	17.01 (1.93)	17.68 (2.25)	16.5 (1.8)
PHB/TB	111 (13)	89.15 (0.55)	−0.85 (0.13)	10.76 (1.79)	10.40 (1.87)	12.7 (0.2)
PLA/PHB	122 (17)	92.60 (0.52)	−1.44 (0.25)	4.97 (0.82)	4.15 (0.78)	18.1 (1.5)
PLA/PHB/TB	114 (9)	90.00 (2.50)	−1.16 (0.25)	7.38 (3.21)	7.01 (3.81)	13.3 (1.9)
PLA/PHB/TB/ChNP	233 (32)	89.00 (1.06)	−1.12 (0.14)	11.82 (1.93)	11.45 (2.15)	12.9 (0.6)

**Table 4:** Thermal characterization data by DSC (first heating scan) and TGA analysis of PLA- and PHB-based materials.

	DSC (I Scan)									TG	DTG	
	T_g,PLA_ [°C]	T_c,PLA_ [°C]	ΔH_c,PLA_ [J g^−1^]	T_m,PLA_ [°C]	ΔH_m,PLA_ [J g^−1^]	T_m,PHB_ [°C]	ΔH_m,PHB_ [J g^−1^]	χ_c,PLA_ [%]	χ_c,PHB_ [%]	T_0_ [°C]	T_max,PHB_ [°C]	T_max,PLA_ [°C]
PLA	62	124	2.5	152	3.3			0.9		322		363
PLA/TB	46	101	25.0	151	25.5			0.6		254		353
PHB						173	85.3		58.4	250	277	
PHB/TB						168	63.9		51.5	210	268	
PLA/PHB	62	122	6.8	152	22.9	171	27.4	28.8	46.8	258	279	351
PLA/PHB/TB	29	77	12.0	146	21.8	165	23.4	20.7	47.2	208	278	358
PLA/PHB/TB/ChNP	27	74	9.1	146	20.1	165	26.0	23.8	53.7	214	277	337

**Table 5:** Mechanical, barrier, and migration behavior of PLA- and PHB-based materials (values in brackets correspond to the standard deviation).

	Mechanical Properties			Barrier Properties			Migration Studies	
	E [MPa]	σ_max_ [MPa]	_εb_ [%]	WVP ∗ 10^11^ [g/s m Pa]	GTR [cm^3^cm/m^2^ d atm]		Isooctane [mg dm^−2^]	Ethanol 10% (*v*/*v*) [mg dm^−2^]
					O_2_	CO_2_		
PLA	2700 (160)	55 (4)	5 (2)	2.2 (0.1)	3.5 (0.1)	4.0 (0.2)	1.7 (0.6)	8.1 (1.8)
PLA/TB	2280 (140)	31 (1)	6 (2)	3.0 (0.1)	4.2 (0.2)	4.3 (0.2)	2.3 (0.9)	14.9 (1.5)
PHB	2230 (130)	34 (2)	4 (1)	0.6 (0.05)	1.1 (0.05)	2.8 (0.1)	2.7 (0.8)	6.7 (0.5)
PHB/TB	880 (50)	15 (1)	2 (1)	2.8 (0.7)	21.1 (0.8)	26.0 (1.0)	5.0 (1.0)	14.5 (0.1)
PLA/PHB	2630 (160)	33 (2)	2 (0)	0.8 (0.1)	1.0 (0.04)	1.6 (0.06)	3.2 (1.6)	7.2 (2.0)
PLA/PHB/TB	960 (60)	12 (1)	67 (6)	2.2 (0.2)	12.6 (0.5)	19.3 (0.7)	8.2 (1.7)	13.7 (2.6)
PLA/PHB/TB/ChNP	2440 (150)	14 (1)	4 (1)	2.9 (0.1)	6.7 (0.3)	17.3 (0.6)	6.7 (2.0)	9.0 (1.8)

## Data Availability

The data presented in this study are available on request from the corresponding author.
